# Bidirectional Association Between Daily Physical Activity and Postconcussion Symptoms Among Youth

**DOI:** 10.1001/jamanetworkopen.2020.27486

**Published:** 2020-11-17

**Authors:** Jingzhen Yang, Menglin Xu, Lindsay Sullivan, H. Gerry Taylor, Keith Owen Yeates

**Affiliations:** 1 Center for Injury Research and Policy, The Abigail Wexner Research Institute at Nationwide Children’s Hospital, Columbus, Ohio; 2 Department of Pediatrics, The Ohio State University, College of Medicine, Columbus; 3 Department of Internal Medicine, The Ohio State University, College of Medicine, Columbus; 4 Discipline of Children's Studies, National University of Ireland, Galway, Galway, Ireland; 5 Biobehavioral Health Center, The Abigail Wexner Research Institute at Nationwide Children’s Hospital, Columbus, Ohio; 6 Department of Psychology, Alberta Children’s Hospital Research Institute, University of Calgary, Calgary, Alberta, Canada; 7 Hotchkiss Brain Institute, University of Calgary, Calgary, Alberta, Canada

## Abstract

This cohort study investigates whether a bidirectional association exists between daily physical activity and postconcussion symptoms among youths with physician-confirmed concussion.

## Introduction

Emerging evidence suggests that youth with concussion who resume physical activity during the acute and subacute phases of injury could have fewer postconcussion symptoms (PCS) and more rapid recovery.^1^ Although earlier physical activity may be associated with fewer PCS, fewer PCS may also be associated with increased activity. Additional research is needed to further our understanding of the directionality of this association. This study investigated the longitudinal and bidirectional association between daily physical activity and PCS during the first week postconcussion among youth aged 11 to 17 years.

## Methods

This cohort study was approved by the Nationwide Children’s Hospital’s institutional review board. Participants provided written informed consent. This study follows the Strengthening the Reporting of Observational Studies in Epidemiology (STROBE) reporting guideline

We prospectively enrolled youth aged 11 to 17 years with a physician-confirmed concussion within 72 hours of injury from the emergency department and concussion clinics at Nationwide Children’s Hospital between [date] and [date].^2^ We measured daily physical activity as reflected in daily step count using an ActiGraph and daily PCS using the Postconcussion Symptom Scale from day 1 to day 7 postinjury.^3^ We grouped daily step count and PCS into 3 waves: days 1 to 3 (wave 1), days 4 to 5 (wave 2), and days 6 to 7 (wave 3) postinjury. Using both a traditional cross-lagged panel model (CLPM) and a random-intercept cross-lagged panel model (RI-CLPM), we examined the bidirectional associations between daily step counts and PCS in this 3-wave, longitudinal design.^4^ RI-CLPM disentangles the within-person process from stable between-person differences, whereas CLPM does not differentiate the covariance between these 2 levels.^4^ Models were estimated using the package lavaan in R statistical software version 3.6.2 (R Project for Statistical Computing), with 2-sided testing and a significance level of α = .05. Statistical analysis was performed from June to July 2020.

## Results

This study’s participants included 83 youth with concussion (54 boys [65%]; mean [SD] age, 14.2 [1.9] years; 59 White participants [72%]; 70 sports-related concussions [84%]). The mean (SD) daily step counts were 9167 (3635) at wave 1, 10 143 (4018) at wave 2, and 10 786 (4038) steps at wave 3, whereas the mean (SD) daily PCS scores were 27.7 (19.6), 21.0 (18.4), and 15.9 (16.4), respectively. Zero-order correlation coefficients between daily step count and PCS were statistically significant (Table). In the CLPM, daily step counts and PCS scores showed significant positive autoregressive associations across all waves (Figure A), reflecting stability across time in both activity and symptoms. In contrast, in the RI-CLPM, which accounted for between-person differences, the only significant autoregressive association was the path for PCS scores from wave 1 to wave 2 (β = 0.652; SE = 0.196; *P* = .002) (Figure B). In the CLPM, only 1 cross-lagged path was significant, with higher PCS scores at wave 1 being associated with lower daily step counts at wave 2 (β = −0.181; SE = 0.101; *P* = .047). No cross-lagged paths were significant in the RI-CLPM. However, daily step counts and PCS showed a significant stable negative correlation between participants (β = −0.454; SE = 0.110; *P* = .03).

**Table.** Zero-Order Correlation Coefficients Between Daily Step Counts and PCS Scores During the First Week Postinjury Among 83 Youth With Concussion Aged 11-17 Years

	Steps, correlation coefficient			PCS score, correlation coefficient		
	1-3 d	4-5 d	6-7 d	1-3 d	4-5 d	6-7 d
Steps and time postinjury						
1-3 d	1	NA	NA	NA	NA	NA
4-5 d	.680^a^	1	NA	NA	NA	NA
6-7 d	.521^a^	.500^a^	1	NA	NA	NA
PCS and time postinjury						
1-3 d	–.402^a^	–.433^a^	–.377^a^	1	NA	NA
4-5 d	–.333^a^	–.422^a^	–.304^a^	.890^a^	1	NA
6-7 d	–.291^b^	–.405^a^	–.346^a^	.818^a^	.831^a^	1

Abbreviations: NA, not applicable; PCS, postconcussion symptom.

^a^ *P* < .01.

^b^ *P* = .01.

**Figure.**
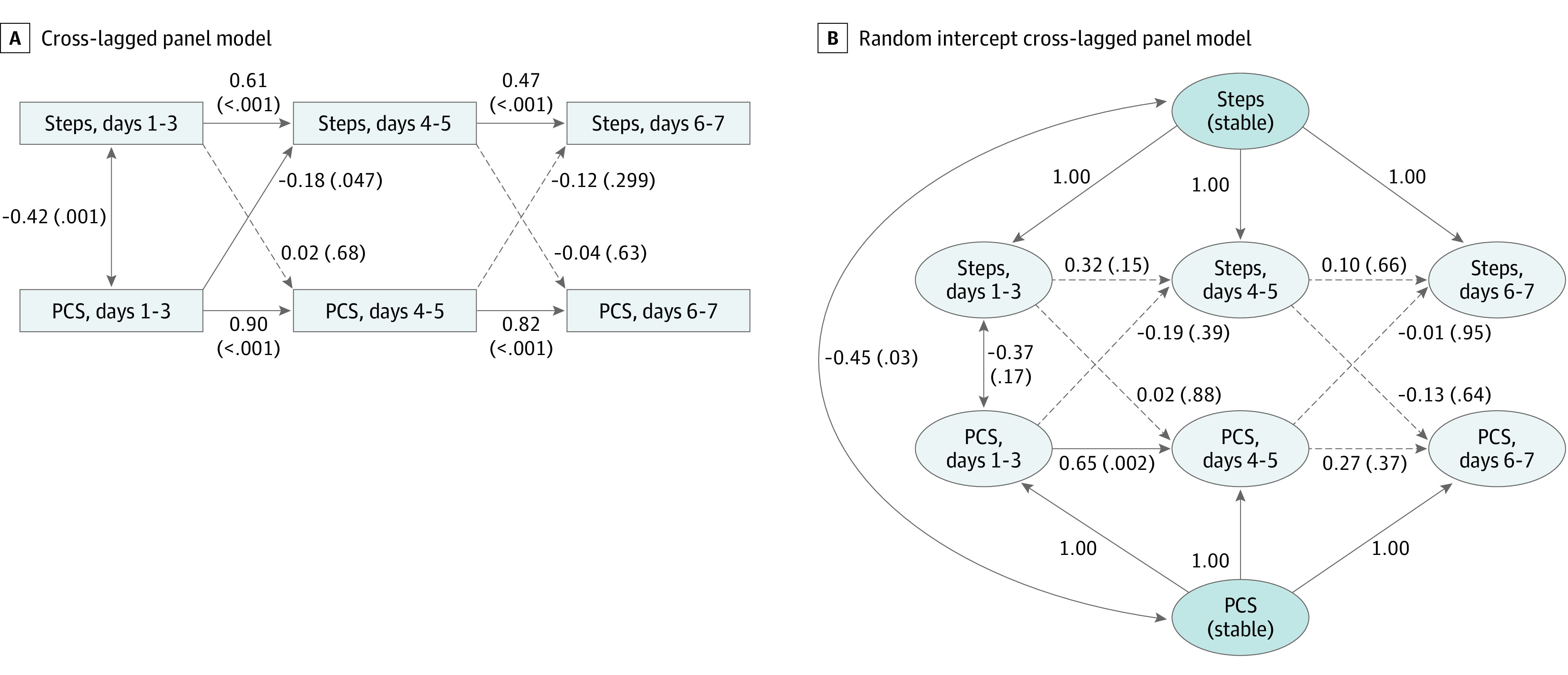
Bidirectional Association of Daily Step Counts and Postconcussion Symptom Scores (PCSs) Among Concussed Youth Aged 11 to 17 Years Diagrams show cross-lagged panel model (A) and random intercept cross-lagged panel model (B) of bidirectional association of daily step counts and PCSs the first week postinjury among 83 concussed youth aged 11 to 17 years. Numbers denote regression coefficients (with *P* values in parentheses).

## Discussion

This study assessed the bidirectional association between physical activity and PCS using cross-lagged panel analyses. Although youth who engaged in more physical activity during the first week postinjury reported fewer PCS than those who engaged in less physical activity, only 1 cross-lagged association between physical activity and PCS was significant. Specifically, greater PCS during wave 1 (days 1 to 3) postinjury was associated with lower daily physical activity during wave 2 (days 4 to 5) postinjury, suggesting that youth with greater PCS may limit their physical activity, perhaps per physician recommendations.^2,5^ However, this association was not apparent in the RI-CLPM, which accounts for between-person associations.

The study was limited by a small sample size and lack of adjustment for individual differences including preinjury physical activity. Future randomized clinical trials with larger sample sizes and longer follow-up are critically needed to better understand the associations of physical activity with PCS and other concussion outcomes among youth.^1,6^ Such studies could inform health care practitioners’ recommendations for physical activity postconcussion and hasten concussion recovery among youth.
